# Beyond the mutation: integrating radiogenomics, epigenetics, and immune signatures to overcome therapeutic resistance in CNS tumors: a narrative review

**DOI:** 10.1097/MS9.0000000000005291

**Published:** 2026-07-15

**Authors:** Shreya Singh Beniwal, Rafael Everton Assunção Ribeiro da Costa, Anushka Anand Hanchate, Chimuka Mwaanga, Elif Özge Çelik, Aarushi Mishra

**Affiliations:** a Lady Hardinge Medical College, Connaught Place, New Delhi, India; b Universidade Estadual de Campinas – Campus Cidade Universitária Zeferino Vaz, São Paulo, Brazil; c Mahatma Gandhi Missions Medical College, Navi Mumbai, Maharashtra, India; d Texila American University Zambia, Lusaka, Zambia; e Turkish Ministry of Health, Van, Turkey; f Danylo Halytskyi Lviv National Medical University, Lviv, Ukraine

**Keywords:** CNS tumors, epigenetics, glioblastoma, immune resistance, radiogenomics

## Abstract

Despite advancements in the field of cancer treatment, its efficacy in tackling central nervous system (CNS) tumors – glioblastomas – remains blunted. This review explores the multifactorial reasons behind these tumors’ resistance to immunotherapeutic strategies, with emphasis on the role of the tumor microenvironment, low mutational burden, and immune exclusion. Emerging immune phenotypes – such as inflamed, excluded, and desert types – have varying contributions to the degree of response. Recent advances in immune profiling, including single-cell RNA sequencing and spatial transcriptomics, have begun to pave the way for a deeper understanding of the immunosuppressive architecture of these tumors, revealing the involvement of tumor-associated macrophages, myeloid-derived suppressor cells, and T-regulatory populations. Additionally, we examine the impact of tumor-nerve crosstalk, T-cell exhaustion, and the limited ability of PD-L1 and tumor-mutation burden as predictive markers. The review also highlights the newfound therapeutic strategies – including CAR-T cell therapy and bispecific antibodies – that aim to overcome immune resistance. By integrating insights from immunogenomics, epigenetics, and radiogenomics, we propose a framework for understanding and potentially reversing immune resistance in CNS tumors. This review highlights the urgent need to develop specific, biomarker-informed approaches that would aim to improve outcomes in this domain of neuro-oncology.

## Introduction

Central nervous system (CNS) tumors include primary gliomas and secondary brain metastases. They exemplify extreme biological complexity and therapeutic resistance. Despite advances in chemotherapeutic, surgical, and radiological interventions, the outcome remains dismal. This is particularly evident in high-grade gliomas, such as glioblastoma (GBM), where median survival barely exceeds 15 months. The inherent heterogeneity of these tumors, spanning intratumoral genomic variability, epigenetic reprogramming, and immune microenvironment dynamics, renders therapeutic resistance not merely a downstream event but a defining characteristic from the outset^[^1,2^]^.

HIGHLIGHTS

- Radiogenomic and epigenetic mechanisms jointly influence therapeutic resistance in central nervous system tumors.
- Immune signatures, including checkpoint modulation and microenvironmental reprogramming, shape treatment outcomes.
- Integration of multi-omic data enhances precision diagnostics and individualized therapy strategies.
- Emerging radiogenomic biomarkers correlate with molecular pathways of resistance.
- Future neuro-oncology requires cross-disciplinary, data-driven therapeutic frameworks.

Traditionally, CNS tumors were classified based on histopathological evidence. However, this paradigm has shifted and has led to the discovery of molecular markers, including isocitrate dehydrogenase (IDH1/2) mutations, 1p/19q co-deletions, and MGMT promoter methylation. This shift has transformed diagnostic and prognostic frameworks. The 2021 WHO CNS tumor classification now mandates integrated molecular subtyping, acknowledging that molecular behavior often supersedes histological appearance in determining clinical trajectory. Despite this evolution, the link between molecular insights and effective treatment remains elusive. Alkylating agents, which were initially effective, are now often outmaneuvered by epigenetic drift and clonal evolution. Additionally, targeted therapies against EGFR or PDGFRA often fail due to bypass signaling, adaptive resistance, or TERT promoter mutations^[^3–5^]^.

Recent findings emphasize the necessity of a systems biology strategy. Radiogenomics – a field that correlates MRI characteristics with tumor genetic and epigenetic signatures – provides a powerful, non-invasive tool to detect spatial heterogeneity and predict treatment response. Simultaneously, single-cell epigenomic analyses uncover dynamic enhancer profiles and DNA methylation plasticity that impart stem-like, therapy-refractory phenotypes. The immunological environment of CNS tumors also has significant resistance: immune privilege, PD-L1-induced T-cell exhaustion, and microglial reprogramming cooperate to suppress immune surveillance, challenging treatment ^[^6–8^]^.

This review suggests a multidimensional approach to understanding therapeutic resistance in CNS tumors. Through the integration of radiogenomic imaging, epigenetic modulation patterns, immune landscape profiling, and NGS-based molecular cartography, we hope to move beyond mutation-focused models. Our goal is to reveal convergent resistance pathways that intersect across tumor subtypes and patient groups. This integrative synthesis is a clinical necessity.

## Methodology

This study was designed as a narrative review with a structured literature search, rather than a formal systematic review. Although elements of the PRISMA 2020 framework were initially considered, the methodological approach aligns more closely with a scoping/narrative synthesis, given the broad and integrative objective of evaluating resistance mechanisms across genomic, epigenomic, immunological, and radiogenomic domains.

A comprehensive literature search was conducted across PubMed, Scopus, and Google Scholar, covering publications from January 2010 to May 2024. Both Medical Subject Headings (MeSH) and free-text terms were employed to maximize sensitivity and specificity. The search strategy combined four conceptual domains:

1. Tumor types: “glioblastoma,” “glioma,” “medulloblastoma,” “ependymoma.”
2. Resistance mechanisms: “immune evasion,” “chemoresistance,” “radioresistance,” “tumor plasticity.”
3. Biomarkers and molecular features: “IDH1,” “MGMT methylation,” “PD-L1,” “TMB,” “ctDNA.”
4. Technologies and approaches: “radiogenomics,” “multi-omics,” “artificial intelligence,” “machine learning.”

Search strings were adapted for each database using Boolean operators (AND/OR) and controlled vocabulary, where applicable.

The inclusion criteria were peer-reviewed articles in English, human studies, original research, systematic reviews, meta-analyses, and studies addressing resistance mechanisms, biomarkers, or multi-omics integration in CNS tumors. The exclusion criteria were non-English publications, animal-only studies, editorials, commentaries, conference abstracts, and articles without accessible full text.

**Figure 1.**
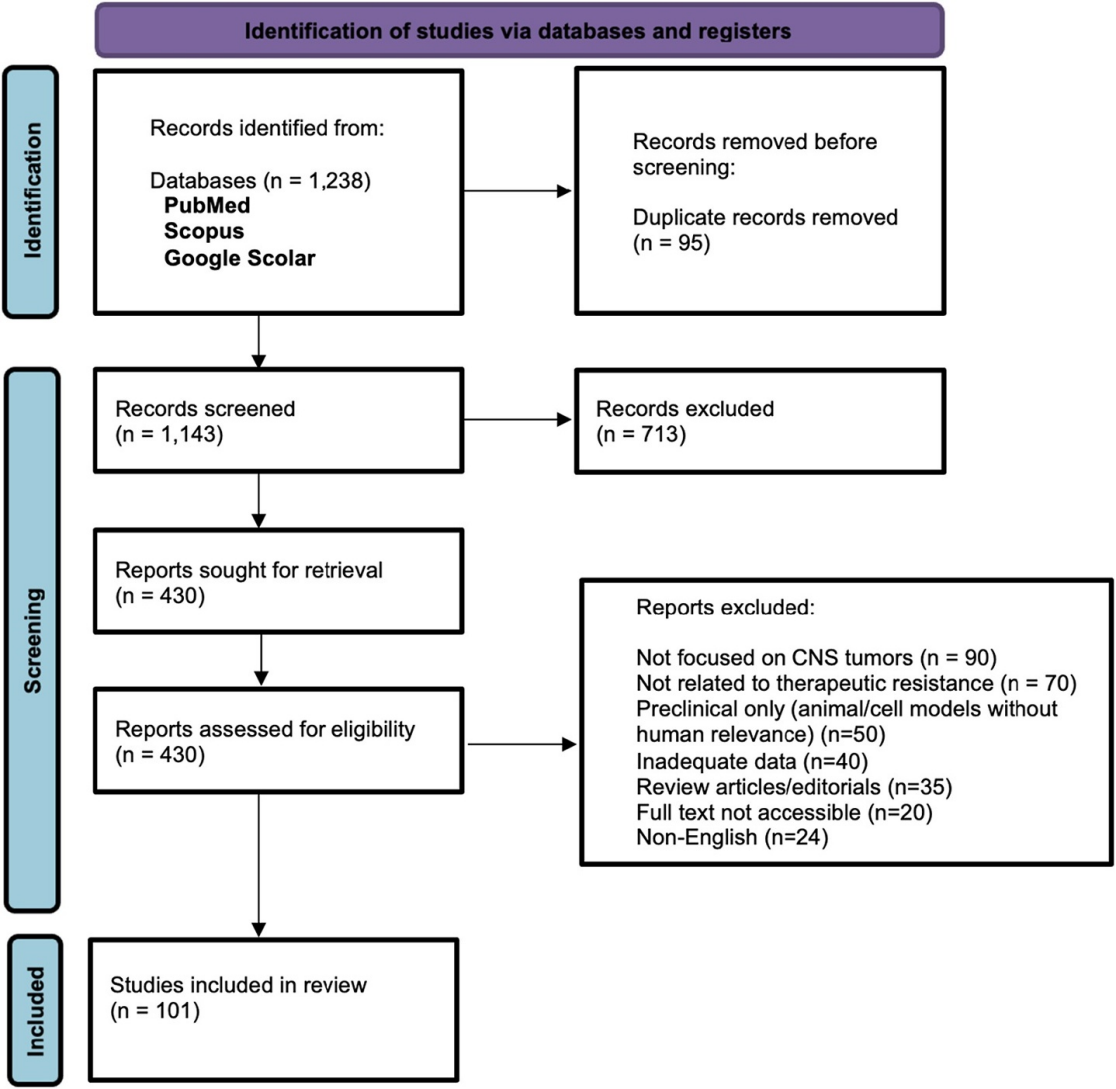
PRISMA 2020 flow diagram of the review.

Study selection was conducted in two stages: (1) title/abstract screening and (2) full-text review. A total of 1238 records were identified, with 95 duplicates removed, resulting in 1143 screened articles. Of these, 430 full-text articles were assessed for eligibility, and 101 studies were included in the final synthesis (Fig. 1). Given the narrative nature of this review, formal quality assessment and risk-of-bias evaluation were not systematically performed, which is consistent with scoping review methodology. Instead, emphasis was placed on the relevance, recency, and methodological robustness of included studies. This approach ensures a robust foundation for the evaluation of therapeutic resistance in CNS tumors across multiple dimensions.

## Genomic and epigenomic drivers of resistance in CNS tumors

CNS tumors, such as GBM and pediatric midline gliomas, exhibit marked resistance to standard treatments, including chemotherapy and radiotherapy. This therapeutic resistance is predominantly driven by adaptive plasticity alongside substantial genomic and epigenomic heterogeneity.

Among the most frequent genetic alterations, IDH1/2 mutations are present in approximately 10% of GBM and lower-grade gliomas. These mutations lead to the production of 2-hydroxyglutarate, which, in turn, causes Glioma CpG Island Methylator Phenotype (G-CIMP). Although G-CIMP is a favorable prognostic indicator in low-grade gliomas, it may lead to secondary oncogenic changes over time^[^9,10^]^. Another important marker is MGMT promoter methylation, which serves as a predictor of temozolomide (TMZ) sensitivity. Nevertheless, resistance frequently develops as a result of re-expression of MGMT or activation of alternative DNA repair pathways^[^11^]^.

EGFR amplifications and EGFRvIII mutations, identified in 30–40% of primary GBM tumors, are known to activate the PI3K/AKT and RAS/MAPK pathways to drive tumorigenicity and resistance to targeted therapy^[^12,13^]^. TERT promoter mutations, which commonly co-occur with ATRX mutations, in turn activate telomerase to achieve and maintain telomere length, as well as permit infinite cellular proliferation. These alterations are associated with poor prognosis and reduced response to standard chemo-radiation therapy^[^14,15^]^. An overview of these genomic and epigenetic alterations, along with their mechanisms of resistance and therapeutic implications, is presented in Table 1.

**Table 1:** Genomic and epigenetic alterations linked to resistance in CNS tumors.

Mutation/modification	Tumor type	Mechanism of resistance	Clinical impact	Therapeutic strategies	Citation
IDH1/2 mutation	Lower-grade gliomas, secondary GBM	Induces G-CIMP phenotype and metabolic reprogramming	Better prognosis and treatment stratification	IDH inhibitors (ivosidenib)	^[^9^]^
EGFR amplification/EGFRvIII	Primary GBM	Activates PI3K/AKT and RAS/MAPK pathways	Poor prognosis; therapy resistance	EGFR-targeted agents	^[^12,13^]^
MGMT promoter methylation	GBM	Impairs DNA repair	Predicts favorable TMZ response	Temozolomide	^[^11^]^
TERT promoter mutation	GBM	Maintains telomere length	Aggressive growth and poor survival	Telomerase inhibitors	^[^14,15^]^
H3K27M mutation	Pediatric diffuse midline gliomas	Epigenetic deregulation	Extremely poor prognosis	EZH2 inhibitors, tazemetostat	^[^16^]^
MSH6 secondary mutation	Recurrent GBM	Mismatch repair loss	Post-TMZ resistance	Immunotherapy	^[^18,19^]^
SMARCA4/SWI-SNF alterations	High-grade gliomas	Disrupted chromatin remodeling	Stem-like resistant phenotype	HDAC inhibitors	^[^17^]^
Global DNA hypo-/hypermethylation	GBM	Tumor suppressor silencing	Treatment resistance	DNA methylation inhibitors	^[^20^]^

H3K27M mutations characterize diffuse midline gliomas in pediatric cases and impede repressive H3K27 trimethylation. This induces epigenetic deregulation, leading to the derepression of oncogenic genes and is associated with extremely aggressive tumor behavior and strong resistance to chemotherapy and radiotherapy. The median survival for these cases is less than 12 months, with H3K27M being the significant resistance driver, and the incidence of secondary mutations, as compared to adult GBM, being lower^[^16^]^.

**Figure 2.**
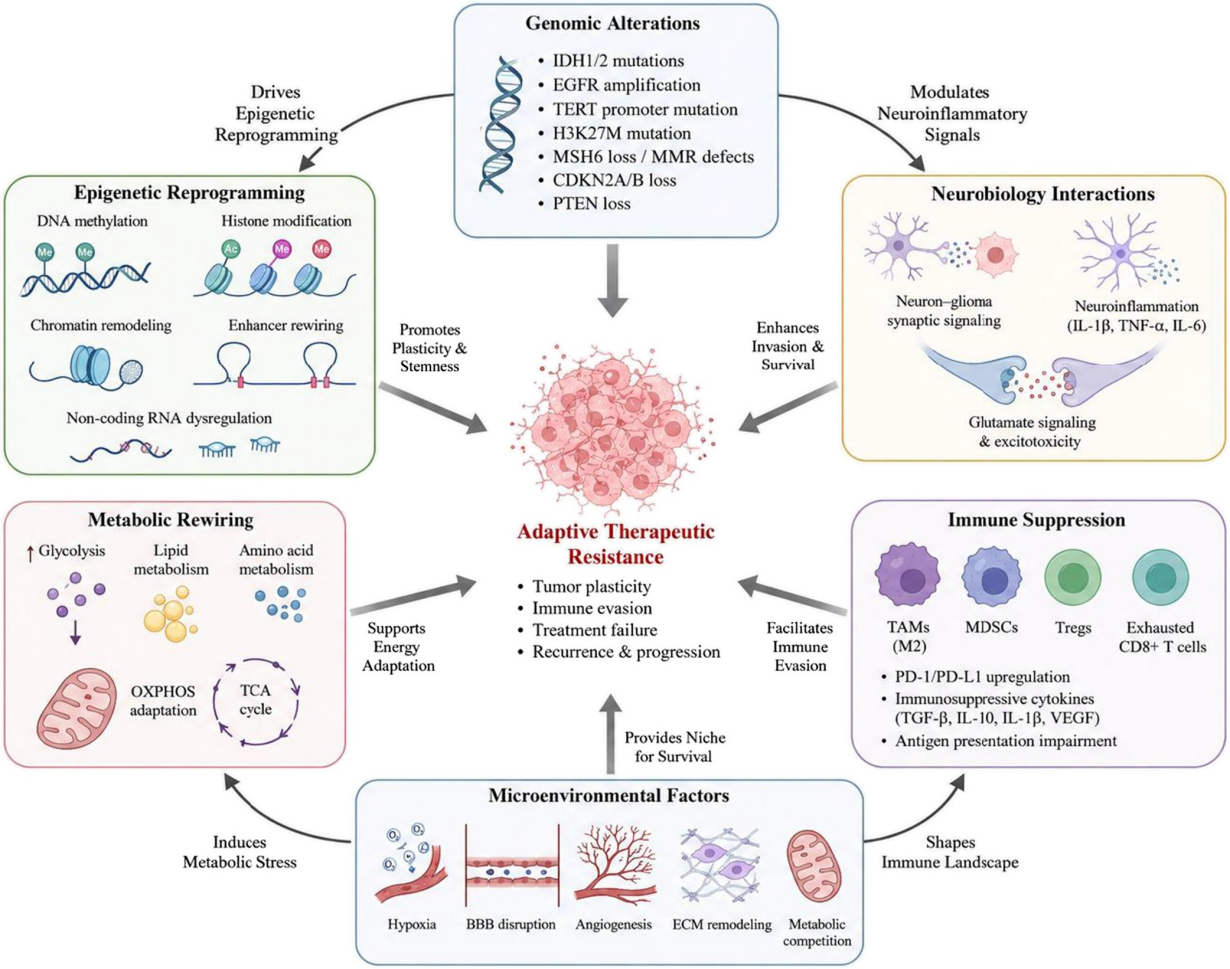
The neuro-immune-epigenetic resistance axis in CNS tumors.

Additionally, they suggest that these miRNAs can be regulators of resistance that is enhanced by epigenetic changes, which are not sequence dependent but still affect gene expression. Global hypermethylation of DNA affects the promoters of tumor suppressor genes, whereas hypomethylation promotes chromosomal instability, with both impacting treatment response. Histone modifications are also important; mutations such as H3K27M decrease H3K27 trimethylation, while HDAC overexpression increases histone acetylation, upregulating BCL2 expression and anti-apoptotic function, leading to resistance. Furthermore, chromatin remodeling is commonly impaired in 20% of cancers due to inactivation of the SWI/SNF complex. In particular, mutations in the SWI/SNF complex2 (SMARCA4) can activate developmental pathways related to the acquisition of stemness characteristics, with the consequence of supplying the material for a stem-cell-like phenotype and thereby contributing to therapy resistance^[^17^]^. The integrated neuro-immune-epigenetic mechanisms driving adaptive therapeutic resistance in CNS tumors are illustrated in Fig. 2^[^9–20^]^.

Secondary mutations can develop as acquired resistance mechanisms after treatment. For instance, MMR mutations (especially MSH6 mutations) may arise after GBM recurrence following TMZ treatment, even though they were not present in pre-treatment specimens. These alterations contribute to a resistant phenotype at recurrence. Prolonged TMZ exposure is also associated with TMZ-induced hypermutation, which is observed in recurrent GBMs and entails C>T/G>A transitions, and it is related to Akt-mTOR and p16/RB pathway mutations. Furthermore, rare secondary mutations in EGFR or IDH1/2 may occur, but their precise mechanisms remain unclear^[^18,19^]^

Molecular profiling for IDH1/2, MGMT methylation, and TERT mutations is now common practice for clinical purposes, contributing to both prognostic and therapeutic decisions. Targeted modalities with drugs such as IDH inhibitors provide encouraging results, especially when personalized with molecular characterization. A new approach to treatment, exemplified for H3K27M-mutant glioma by tazemetostat, involves epigenetic modulators, while EGFR-directed therapies continue to encounter resistance. Additionally, serial profiling of relapse tumors for rising mutations, such as MSH6 or hypermutator signatures, is becoming more crucial for treatment decisions. Highly mutated, MMR-deficient gliomas were approached by a combinatorial strategy in which TMZ was combined with DNA repair inhibitors or immunotherapies, which may ultimately enhance clinical outcomes in MMR-deficient, hypermutated gliomas^[^21–23^]^.

## Immune profiling and failure of immunotherapy

Immunotherapy has revolutionized cancer care, yet its success in CNS tumors – namely GBM – has been limited, with response rates often falling below 10% and survival rarely exceeding 15 months^[^24–26^]^. GBMs are the most aggressive primary brain tumors, and they present unique challenges that limit the effectiveness of immunotherapy. These challenges arise from a unique tumor microenvironment (TME) that is marked by low tumor-mutation burden (TMB), poor immunogenicity, extensive inter- and intra-tumoral heterogeneity, as well as immune exclusion^[^24,25^]^. Additionally, the BBB restricts immune cell infiltration and therapeutic antibody delivery, thereby reducing CD8+ T-cell infiltration and checkpoint inhibitor efficacy in CNS tumors^[^24^]^.

CNS tumors can be phenotypically categorized into inflamed, excluded, and desert subtypes, with each exhibiting varying degrees of responsiveness to immune checkpoint inhibitors^[^26–28^]^ – the inflamed subtype often demonstrates the most favorable outcomes. GBM typically shows an immune desert phenotype with abundant tumor-associated macrophages (TAMs) and myeloid-derived suppressor cells (MDSCs) but minimal T-cell infiltration, while brain metastases, on the other hand, display a more inflamed TME that is rich in CD8+ T cells and T-regulatory (Treg) cells, which are accompanied by higher PD-L1 expression and disrupted BBB integrity^[^19,21–26^]^. Multiple factors contribute to this classification, including tumor genetics, immune remodeling, and metabolic adaptations, including tumor genetics, immune cell remodeling, and cancer metabolism^[^28^]^. Each phenotype exhibits a distinct escape mechanism, identified using multi-omics platforms such as single-cell RNA sequencing (scRNA-seq), CyTOF, and spatial transcriptomics^[^28^]^.

T-cell exhaustion – marked by continuous expression of inhibitory receptors such as PD-1, CTLA-4, and TIM3 – is a major contributor to immune dysfunction in CNS tumors, with evidence emerging that indicates distinct exhaustion phenotypes in GBMs^[^29^]^. Several biomarkers have been investigated to predict response to immune checkpoint inhibitors (ICIs), including PD-L1 expression, TMB, and T-cell inflamed gene expression signatures^[^30^]^. Although TMB and T-cell inflamed gene expression profiles have demonstrated predictive value in extracranial tumors^[^30^]^, in CNS tumors, PD-L1 and TMB frequently show limited correlation and weak predictive power^[^31^]^. Interestingly, in estrogen receptor-positive breast cancers, some patients have shown response to ICIs despite being PD-L1 negative, suggesting that exhausted CD8+ T cells (CD8+ PD-1 +/CTLA-4 +) and Treg depletion could all serve as predictive markers – a possible approach worth exploring in CNS tumors^[^32^]^.

Advanced platforms like single-cell immune profiling, T-cell receptor barcoding, and spatial transcriptomics have facilitated the identification of immune-responsive subpopulations in gliomas and brain metastases^[^33,34^]^. Myeloid cell-related transcriptional signatures, including TAMs and myeloid-derived suppressor cells (MDSCs), are being recognized as possible biomarkers that influence therapy outcomes^[^35^]^. Technologies such as single-cell RNA sequencing, mass cytometry, and spatial transcriptomics provide deeper insight into tumor-immune dynamics, which contribute to advancing biomarker discovery^[^36^]^. Both primary and secondary acquired resistance in GBM are increasingly associated with immunosuppressive phenotypes – such as macrophage polarization and reduced T-cell clonality – emphasizing the need for further immunogenomic characterization^[^37^]^.

**Figure 3.**
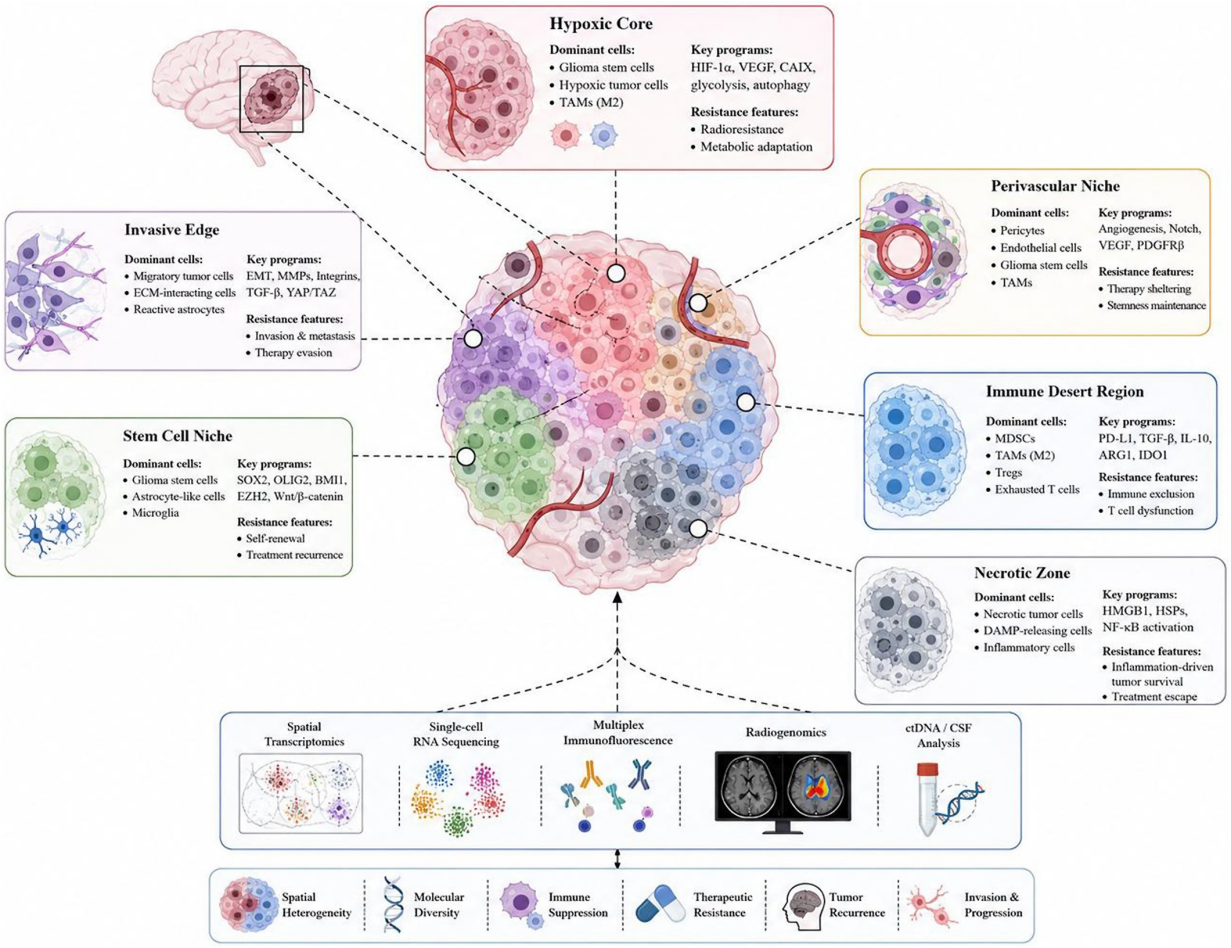
Spatial resistance niches and cellular ecosystems in CNS tumors.

CNS tumors’ microenvironment plays an integral role in mediating resistance to immunotherapy, mainly through the accumulation of immunosuppressive myeloid cells. TAMs, MDSCs, and Tregs all dominate the CNS TME; they contribute to immune evasion by secreting immunosuppressive cytokines such as IL-1 and TGF-β, in addition to expressing PD-L1^[^38^]^. The link between tumor-derived growth factors and the immunosuppressive cell populations further amplifies T-cell dysfunction, promoting an environment that protects tumor cells from immune surveillance^[^39^]^. The spatial organization of resistance niches and cellular ecosystems contributing to therapeutic resistance in CNS tumors is illustrated in Figure 3^[^24,26–28,33–41^]^.

Brain metastases differ from primary gliomas by exhibiting reduced CD8+ T-cell infiltration and altered PD-1/PD-L1 dynamics^[^40^]^. This highlights the need for sub-type specific therapeutic strategies and improved visual stratification. Additionally, the criteria that exclude these patients from clinical trials further limit available data and the subsequent development of better and more effective therapeutic strategies^[^40^]^. Emerging strategies – such as biomarker-guided patient selection, bispecific antibodies targeting EGFR and PD-1, as well as CAR-T therapies (e.g., EGFRvIII-directed) – are being evaluated as potential approaches to overcome therapeutic resistance in CNS tumors^[^40^]^.

Both tumor cell plasticity and cancer stem-like cells promote immune evasion, which contributes to therapeutic resistance through certain mechanisms, such as antigen loss, enhanced DNA repair capabilities, and upregulation of immune checkpoint molecules^[^41–43^]^. In addition, various microenvironment barriers, such as altered neurovascular permeability and extracellular matrix remodeling, among others, further exacerbate resistance^[^24^]^. Tumor-nerve interactions have recently been implicated in promoting immune evasion in gliomas via signaling pathways that influence immune cell trafficking and cytokine selection – a potentially new therapeutic axis^[^41^]^.

In conclusion, immunotherapy has brought about major advances in cancer treatment, but CNS tumors continue to present unique challenges hindering its effectiveness. The complex TME and the various resistance mechanisms render responses to therapy inconsistent and ineffective. Given the limitations of PD-L1 and TMB as standalone predictors of response, integrated multi-parameter frameworks – incorporating spatial transcriptomics, immune deconvolution, and myeloid cell signatures – may provide more accurate insights into CNS immunotherapy^[^24,42,43^]^.

## Radiogenomic correlations

Radiogenomics is a novel scientific field that integrates knowledge from medical imaging (radiomics) with information on individual gene expression patterns to support the diagnosis, prognosis, and treatment of diseases such as cancer. AI, in turn, encompasses machine learning and deep learning approaches that have been increasingly used in the field of radiogenomics, as it enables the extraction of complementary information from complex datasets found in large clinical and imaging databases^[^44^]^.

In neuro-oncology, AI is increasingly applied to MRI interpretation in order to identify tumor margin infiltration, distinguish between pseudoprogression and true tumor progression, predict recurrence and survival information, and non-invasively detect genetic mutations and epigenetic alterations, such as *IDH* (isocitrate dehydrogenase) and *MGMT* (O6-methylguanine-DNA methyltransferase) genes. Therefore, AI-based radiomics and radiogenomics expand the information available on diagnosis, staging, genetic mutation status, aggressiveness, therapeutic response, recurrence, and survival, allowing for better clinical management of brain tumor cases^[^44,45^]^.

Imaging data can be employed for the quantitative assessment of biomarkers through a structured sequence of processing steps that allows for segmentation and visualization of the lesion. Subsequently, AI algorithms are applied to extract quantitative features such as morphology, volumetric parameters, intensity-based metrics, and statistical descriptors derived from image analysis, including histogram-based features, texture features, biophysical model fitting, spatial pattern recognition, and deep learning-derived features. This radiomics-driven approach facilitates the development of clinically relevant insights, such as the inference of underlying molecular profiles^[^46^]^.

The genetic and molecular characterization of CNS tumors has advanced substantially in recent years. For instance, it is now well established that mutations in the *IDH* and *MGMT* genes are associated with more favorable prognoses in GBM, the most common primary brain tumor. Recent studies have identified qualitative MRI features predictive of *IDH* mutation in GBM, including frontal lobe predominance, absence or minimal contrast enhancement, smaller tumor size, and sharply demarcated margins. In the context of *MGMT*, associated imaging characteristics may include frontal lobe localization (often overlapping with *IDH*-mutant lesions), the presence of an eccentric necrotic cyst, and elevated apparent diffusion coefficient values^[^47^]^.

In the context of brain tumors, preliminary studies have described analytical models based on MRI imaging features such as enhancement (signal intensity of brain tissue after contrast administration), diffusion (a method that generates images of biological tissues based on local microstructural characteristics of water diffusion), and perfusion (measurement of blood flow), which are associated with the presence of the following molecular markers: *IDH, TERT* (telomerase reverse transcriptase) and *EGFR* (epidermal growth factor receptor) mutations, + 7/ − 10 copy number alteration, 1p/19q co-deletion, H3 K27-altered, and H3 G34-mutant^[^48–50^]^.

Current evidence on radiogenomic markers involved in brain tumor progression indicates that upregulation of the *PERIOSTIN* (*POSTN*) gene and downregulation of miR-219 are associated with shorter progression-free survival. Conversely, patients with higher levels of neuronal N-acetylaspartate (nNAA) tend to exhibit longer progression-free survival. The presence of *IDH* and *MGMT* mutations is linked to reduced tumor progression, whereas mutations in *NF1* (neurofibromatosis type 1), *PTEN* (phosphatase and tensin homolog), *EGFR* (epidermal growth factor receptor), *TP53* (tumor protein p53), *NOTCH2* (neurogenic locus notch homolog protein 2), and *GIGYF2* (GRB10 interacting GYF protein 2) may be associated with increased tumor progression^[^51,52^]^.

## Next-generation sequencing and cerebrospinal fluid–based liquid biopsies in CNS tumors: diagnostic and monitoring advances

Next-generation sequencing (NGS) has significantly advanced the molecular profiling of CNS tumors by enabling the high-throughput detection of a broad range of genomic alterations. These include single nucleotide variants (SNVs), copy number variations (CNVs), and gene fusions, all of which are critical for accurate tumor classification, prognostication, and treatment planning. In gliomas, for instance, NGS facilitates the identification of clinically actionable mutations such as IDH1/2 SNVs, EGFR amplifications (a form of CNV), and gene fusions such as FGFR-TACC, all of which contribute to the development of personalized therapeutic strategies^[^53,54^]^. The ability of NGS to detect multiple genetic alterations in a single assay presents a significant advantage over traditional single-gene testing approaches^[^55^]^.

Despite the utility of tissue biopsy as the current gold standard for diagnosis, it is associated with several limitations, particularly in CNS tumors. Tissue biopsies are invasive and carry procedural risks, especially when tumors are located in surgically inaccessible or high-risk regions. Additionally, tumor heterogeneity and limited sampling can lead to inaccurate molecular characterization and an incomplete understanding of disease progression^[^56^]^. In this context, cerebrospinal fluid (CSF)-based liquid biopsy has emerged as a promising, minimally invasive alternative. CSF is in direct contact with CNS tumors, making it a more representative biological source of tumor-derived genetic material than plasma. Furthermore, CSF sampling via lumbar puncture allows for repeated molecular analysis over time, supporting longitudinal disease monitoring^[^57,58^]^.

Among the most valuable analytes identified through CSF-based liquid biopsy are circulating tumor DNA (ctDNA) and microRNAs (miRNAs). ctDNA provides a real-time molecular snapshot of tumor-specific genomic alterations and is typically found in higher concentrations in CSF than in plasma, where it is diluted by systemic circulation^[^59^]^. Similarly, miRNAs such as miR-21 and miR-10b have been associated with tumor aggressiveness and progression in CNS malignancies. These CSF-derived miRNAs exhibit superior specificity compared to plasma-based counterparts, underscoring their potential as robust biomarkers for diagnosis and disease monitoring^[^60,61^]^.

**Figure 4.**
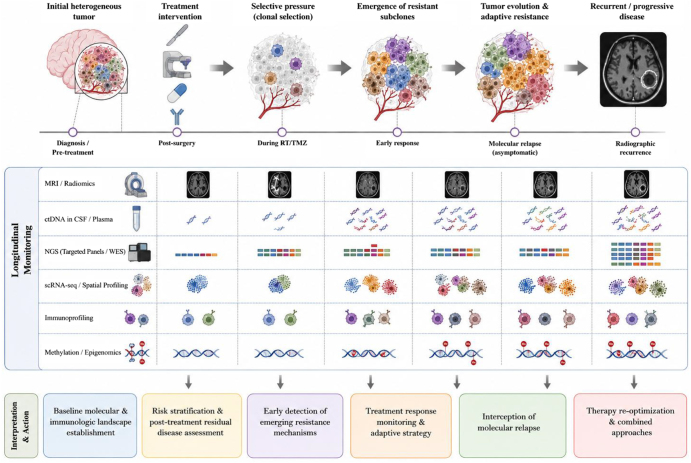
Spatiotemporal evolution of resistance and liquid biopsy monitoring in CNS tumors.

Importantly, integrating NGS with CSF-derived ctDNA enables dynamic monitoring of therapeutic response and the early detection of resistance mechanisms. Fluctuations in ctDNA levels have been shown to correlate more closely with tumor burden than conventional imaging modalities, allowing for earlier intervention and more timely adjustments in treatment^[^62^]^. Longitudinal sequencing can identify the emergence of resistance mutations – such as secondary EGFR alterations – facilitating informed therapeutic decision-making in real time^[^63^]^. The spatiotemporal evolution of therapeutic resistance and longitudinal liquid biopsy monitoring in CNS tumors are illustrated in Figure 4 ^[^56–64^]^. Although challenges remain in terms of sensitivity and sample quality, ongoing advances in sequencing technologies and bioinformatic tools continue to enhance the clinical reliability and utility of CSF-based liquid biopsy^[^64^]^.

## Future directions: a precision resistance map

Multi-omics integration enables a comprehensive understanding of CNS tumor biology by capturing molecular alterations across genomic, epigenomic, transcriptomic, proteomic, and metabolomic levels. Integrating genomic and epigenomic datasets can identify mutations, methylation signatures, and copy-number variations that drive tumor progression and therapeutic resistance. Meta-analyses performed by Maiorano *et al* and Liu *et al*^[^65,66^]^ emphasized that AI-assisted next-generation sequencing approaches – including whole-exome sequencing (WES), whole-genome sequencing (WGS), RNA sequencing, miRNA profiling, and methylation analysis – can stratify CNS tumors according to actionable molecular alterations while simultaneously uncovering hidden resistance mechanisms.

Recent advances in radiogenomics have further strengthened the integration of imaging and molecular oncology. Tan *et al*, Wang *et al*, Sharma *et al*, and Zhang *et al*^[^67–70^]^ demonstrated that AI-enhanced radiogenomics can non-invasively infer molecular tumor profiles and predict resistance-associated gene-expression patterns using MRI-derived imaging signatures. These approaches may facilitate dynamic therapeutic monitoring and individualized treatment adaptation in CNS tumors.

Prediction of treatment escape through liquid biopsy and longitudinal surveillance has emerged as another promising strategy for overcoming therapeutic failure. Zhang *et al* demonstrated that CSF-derived circulating tumor DNA (ctDNA) provides a minimally invasive and dynamic representation of the evolving tumor genome, enabling the detection of treatment-evasive mutations and clonal evolution during disease progression^[^71^]^.

Artificial intelligence methodologies – particularly machine learning (ML) and deep learning (DL) – have become increasingly important for the interpretation of high-dimensional multi-omics datasets in neuro-oncology. Zhan *et al*, Cè *et al*, and Cao *et al*^[^72–74^]^ demonstrated that AI-assisted analytical frameworks can improve glioma subtype classification, prognostic prediction, treatment-response assessment, and clinical decision-making. These approaches can identify nonlinear biological interactions and latent molecular patterns that are often undetectable using conventional statistical methods.

Emerging therapeutic strategies targeting resistance-associated pathways have also gained considerable attention. Li *et al* demonstrated that modulation of the lncRNA SNHG15/CDK6/miR-627 signaling axis may overcome TMZ resistance while reducing M2 polarization of glioma-associated microglia^[^75^]^. Similarly, Gao *et al* highlighted the importance of EGFR-activated metabolic pathways in GBM resistance biology and identified these pathways as potential therapeutic targets^[^76^]^.

Radiogenomic integration studies performed by He *et al* demonstrated that quantitative imaging phenotypes can be directly linked to genomic and transcriptomic alterations, thereby strengthening precision-oncology frameworks in CNS tumors^[^77^]^. Feng *et al* further demonstrated the emerging therapeutic relevance of EGFR tyrosine kinase inhibitors (EGFR-TKIs) in treatment-resistant brain tumors and emphasized their potential future applications in GBM management^[^78^]^. Lee *et al* subsequently showed that radiomics and artificial intelligence models significantly improve molecular classification and prognostic stratification in brain tumors^[^79^]^.

Despite these advances, immunotherapy efficacy in GBM remains limited because of profound immune evasion and marked intratumoral heterogeneity. Zhang *et al* demonstrated that current immunotherapeutic approaches in GBM continue to face substantial limitations related to immune suppression and therapeutic resistance^[^80^]^.

Ozaki *et al* systematically reviewed the application of AI-integrated multi-omics approaches in cancer diagnosis and prognosis and demonstrated that combining genomics, transcriptomics, epigenomics, and proteomics substantially improves diagnostic precision and prognostic stratification^[^81^]^. Multi-omics frameworks may, therefore, provide deeper insights into resistance evolution and tumor adaptation in CNS malignancies.

Pennisi *et al* demonstrated the therapeutic relevance of telomerase-targeted strategies in GBM^[^82^]^. Yan *et al* further showed that CD73-mediated A2B adenosine receptor signaling promotes GBM progression and chemoresistance^[^83^]^. Ferri *et al* highlighted the importance of targeting DNA-damage-response pathways to overcome therapeutic resistance in GBM^[^84^]^. Similarly, Ezzati *et al* reviewed the current status and future potential of EGFR inhibitors in GBM management^[^85^]^.

Advanced radiomics methodologies continue to expand the role of artificial intelligence in neuro-oncology imaging. Zilka *et al* demonstrated that computer-vision–based radiomics approaches improve tumor characterization and imaging analytics in CNS neoplasms^[^86^]^.

Epigenetic dysregulation also plays a major role in treatment resistance. Xie *et al* demonstrated that DNA methylation and histone modifications significantly contribute to GBM resistance mechanisms and may serve as important therapeutic targets^[^87^]^. Li *et al* further showed that immune escape and intratumoral heterogeneity remain major contributors to immunotherapy resistance in CNS tumors^[^88^]^.

Liquid-biopsy–based longitudinal monitoring continues to evolve rapidly in neuro-oncology. Seyhan AA highlighted the emerging importance of circulating liquid-biopsy biomarkers for the dynamic monitoring of GBM evolution, treatment response, and resistance development^[^89^]^.

Recent large-scale systematic reviews have demonstrated the growing clinical utility of artificial intelligence in neuro-oncology prognostication. Mohammadzadeh *et al* showed that AI-assisted survival-prediction models significantly improve outcome prediction in high-grade gliomas^[^90^]^. Pacchiano *et al* further demonstrated the expanding applications of radiomics and artificial intelligence in pediatric brain tumors^[^91^]^.

Novel therapeutic strategies targeting glioma stem cells and resistance-associated tumor subpopulations are also gaining increasing attention. Smiley *et al* demonstrated that CD133-targeted polymeric nanoparticles may improve therapeutic delivery to GBM stem cells and potentially overcome resistance mechanisms^[^92^]^. Earlier work by Haar *et al* additionally established that GBM resistance is driven by complex interactions involving tumor stemness, DNA repair, metabolic adaptation, and TME signaling^[^93^]^.

Artificial intelligence–based multi-omics integration platforms are increasingly fueling precision oncology by combining molecular profiling, imaging, and longitudinal patient datasets into unified predictive frameworks. He *et al* demonstrated that AI-assisted multi-omics integration substantially improves cancer precision-medicine strategies^[^94^]^. Bakas *et al* subsequently proposed the AI-RANO framework to improve standardization, validation, and reproducibility of AI-assisted neuro-oncology response assessment^[^95^]^.

Recent advances in GBM immunotherapy continue to reveal both opportunities and limitations associated with immune-based treatment strategies. Maccari *et al* highlighted the current challenges and future directions of GBM immunotherapy^[^96^]^. De Bonis *et al* further reviewed the role and limitations of anti-angiogenic therapies in high-grade gliomas^[^97^]^. Similarly, Shukla *et al* emphasized the therapeutic significance of cancer stem cell-targeting approaches in translational oncology^[^98^]^.

Standardization of radiomics methodologies has become increasingly important for ensuring reproducibility and reliability across AI-based neuro-oncology studies. Whybra *et al* proposed standardized radiomics convolutional-filter frameworks to improve reproducibility and facilitate the clinical translation of imaging biomarkers^[^99^]^. Finally, Liu *et al* demonstrated the expanding role of AI-based radiogenomics frameworks in complex neurological diseases, further supporting the future integration of dynamic AI-assisted resistance prediction models in CNS tumors^[^100^]^. Table 2 highlights the expanding role of AI-integrated multi-omics platforms in decoding therapeutic resistance across CNS tumors. By integrating radiomics, genomics, epigenomics, liquid-biopsy biomarkers, and explainable AI frameworks, these approaches enable real-time resistance prediction, molecular stratification, and adaptive precision-therapy modeling, thereby advancing next-generation neuro-oncology care^[^65–100^]^.

**Table 2:** Epigenetic mechanisms driving resistance in glioblastoma.

Epigenetic mechanism	Molecular target/process	Impact on resistance	Citation
DNA methylation	MGMT promoter, CpG island hypermethylation	Silencing of DNA repair genes (e.g., MGMT) reduces temozolomide efficacy and suppresses tumor suppressor pathways	^[^11,87^]^
Histone modifications	H3K27me3, H3K9ac, H4 acetylation/methylation	Alters chromatin accessibility, supports glioma stem-like cell survival, and promotes phenotypic plasticity	^[^16,87^]^
Non-coding RNAs (miRNAs, lncRNAs)	miR-21, HOTAIR, SNHG15	Regulate apoptosis, stemness, immune evasion, and DNA repair pathways; epigenetically reprogram resistance networks	^[^11,75,87^]^
Chromatin remodeling	EZH2, SWI/SNF complex alterations	Facilitates transcriptional reprogramming and maintenance of therapy-resistant tumor subpopulations	^[^17,87^]^
Epigenetic plasticity	Proneural-to-mesenchymal transition and adaptive state switching	Enables tumor adaptation under therapeutic stress and contributes to recurrence after treatment	^[^87,93^]^

## Current limitations

Despite major advances in molecular profiling and precision neuro-oncology, several limitations continue to hinder therapeutic success in glioblastoma and other CNS tumors. One of the major barriers is the profoundly immunosuppressive TME, characterized by the accumulation of regulatory T cells (Tregs), TAMs, and myeloid-derived suppressor cells (MDSCs), which collectively inhibit cytotoxic T-cell activation and antigen presentation^[^80,88,96^]^. This immunosuppressive milieu substantially limits the efficacy of immune-checkpoint inhibitors and other immunotherapeutic strategies^[^80,88,96^]^.

Another major challenge is extensive intratumoral heterogeneity. CNS tumors frequently contain multiple genetically and phenotypically distinct subclonal populations that evolve dynamically under therapeutic pressure. Such heterogeneity contributes to variable neoantigen expression, adaptive resistance mechanisms, and inconsistent therapeutic responses^[^87,88,93^]^. Furthermore, glioma stem-cell populations exhibit enhanced resistance to chemotherapy and radiotherapy through activation of DNA-repair pathways, stemness-associated signaling networks, and metabolic reprogramming^[^82–84,92,93,98^]^.

The BBB also significantly restricts the penetration of monoclonal antibodies, CAR-T cells, and many systemic therapeutics into tumor tissue, thereby limiting effective drug delivery^[^80,96^]^. In addition, variability in imaging protocols, radiomics pipelines, and multi-omics integration methods continues to impair the reproducibility and generalizability of artificial intelligence models in neuro-oncology^[^79,94,95,99^]^.

Although AI-integrated multi-omics approaches demonstrate considerable promise, many studies remain retrospective or exploratory in nature and are frequently limited by small sample sizes, a lack of external validation, and insufficient standardization^[^90,94,95,99^]^. Collectively, these biological, technological, and translational barriers continue to limit durable therapeutic responses in CNS tumors and highlight the need for integrated multimodal therapeutic strategies.

## Challenges and future directions

Future progress in CNS tumor management will depend heavily on the successful integration of artificial intelligence, radiogenomics, immunogenomics, and multi-omics precision-oncology frameworks into routine clinical practice^[^72–81,94,95^]^. However, several major challenges remain. One of the primary limitations involves the high dimensionality of omics datasets relative to comparatively limited patient sample sizes, substantially increasing the risk of model overfitting and reduced generalizability^[^81,90,94^]^.

Another major issue involves the lack of standardization across data acquisition, preprocessing, radiomics feature extraction, sequencing methodologies, and computational pipelines. Differences in imaging platforms, MRI acquisition parameters, and molecular-analysis workflows may significantly affect the reproducibility of AI-generated predictions^[^79,95,99^]^. Therefore, the development of harmonized multicenter datasets and standardized analytical frameworks remains essential for reliable clinical translation^[^95,99^]^.

Interpretability of deep-learning systems also remains a major challenge. Many AI algorithms continue to function as “black-box” models, limiting clinician confidence and reducing transparency regarding the biological rationale underlying predictions^[^72,74,94^]^. Integration of explainable artificial intelligence (XAI) methodologies – including SHAP, LIME, and interpretable neural-network architectures – may improve physician trust and facilitate clinical adoption of AI-assisted decision-support systems^[^74,94,95^]^.

Future neuro-oncology research will likely emphasize longitudinal and adaptive precision-oncology frameworks capable of continuously integrating imaging, molecular profiling, liquid-biopsy monitoring, and immune-landscape characterization^[^71,89,94,95^]^. Such systems may allow early prediction of therapeutic resistance, optimization of treatment sequencing, and identification of transient therapeutic vulnerabilities during tumor evolution^[^87,90^]^.

Combination therapeutic strategies are also expected to become increasingly important. Integration of immunotherapy with radiotherapy, anti-angiogenic agents, epigenetic therapies, stem-cell–targeted approaches, and molecular inhibitors may improve treatment efficacy while delaying adaptive resistance^[^82–98^]^. Simultaneously, AI-assisted predictive modeling may facilitate patient stratification and identification of individuals most likely to benefit from specific therapeutic combinations^[^72–81,90,94^]^.

Federated-learning approaches and privacy-preserving AI frameworks may further support large-scale collaborative neuro-oncology research while maintaining patient confidentiality^[^94,95^]^. Ultimately, integration of AI-driven multi-omics profiling, radiogenomics, longitudinal liquid-biopsy surveillance, and adaptive therapeutic modeling may establish truly personalized resistance-mapping frameworks capable of substantially improving outcomes in CNS tumors^[^65–100^]^.

## Conclusion

Therapeutic resistance in CNS tumors, particularly glioblastoma, remains a formidable clinical challenge driven by complex, overlapping mechanisms. Traditional approaches targeting single genetic alterations are insufficient in addressing tumor heterogeneity, adaptive plasticity, and an immunosuppressive microenvironment. This review highlights the necessity of a multidimensional model that integrates genomic, epigenetic, immunological, and radiological data. Radiogenomics enables non-invasive prediction of molecular subtypes, while epigenetic profiling reveals dynamic reprogramming mechanisms that sustain tumor stemness and therapy evasion. Meanwhile, immune signatures provide insights into immunotherapy resistance. Liquid biopsies and next-generation sequencing further empower longitudinal monitoring of resistance mutations.

The integration of these domains, augmented by AI and machine learning, offers a robust framework to anticipate resistance trajectories and guide adaptive treatment strategies. Emerging evidence supports the use of AI-driven platforms for patient stratification, resistance prediction, and real-time therapeutic decision-making. However, challenges such as data standardization, model interpretability, and clinical validation must be addressed to ensure clinical translatability into routine practice. Ultimately, the future of CNS tumor management lies in precision resistance mapping, which represents an integrative, systems-level approach capable of personalizing therapy, prolonging response durations, and overcoming the adaptive resilience of these malignancies. Translating this vision into clinical reality will require coordinated efforts across disciplines, prospective multi-institutional studies, and the development of suitable AI frameworks.

## Data Availability

All data analyzed in this study were obtained from previously published articles and publicly available sources cited within the reference list.

## Research registration unique identification number (UIN)

Not applicable. This study is a narrative review based exclusively on previously published literature and, therefore, did not require prospective registration.
